# Red oak seedlings as indicators of deer browse pressure: Gauging the outcome of different white‐tailed deer management approaches

**DOI:** 10.1002/ece3.5729

**Published:** 2019-11-08

**Authors:** Bernd Blossey, Paul Curtis, Jason Boulanger, Andrea Dávalos

**Affiliations:** ^1^ Department of Natural Resources Cornell University Ithaca NY USA; ^2^ University of North Dakota Grand Forks ND USA; ^3^ Biological Sciences SUNY Cortland Cortland NY USA

**Keywords:** deer management, forest regeneration, oak browse rate, *Odocoileus virginianus*, *Quercus rubra*, recreational hunting

## Abstract

After decades of high deer populations, North American forests have lost much of their previous biodiversity. Any landscape‐level recovery requires substantial reductions in deer herds, but modern societies and wildlife management agencies appear unable to devise appropriate solutions to this chronic ecological and human health crisis. We evaluated the effectiveness of fertility control and hunting in reducing deer impacts at Cornell University. We estimated spring deer populations and planted *Quercus rubra* seedlings to assess deer browse pressure, rodent attack, and other factors compromising seedling performance. Oak seedlings protected in cages grew well, but deer annually browsed ≥60% of unprotected seedlings. Despite female sterilization rates of >90%, the deer population remained stable. Neither sterilization nor recreational hunting reduced deer browse rates and neither appears able to achieve reductions in deer populations or their impacts. We eliminated deer sterilization and recreational hunting in a core management area in favor of allowing volunteer archers to shoot deer over bait, including at night. This resulted in a substantial reduction in the deer population and a linear decline in browse rates as a function of spring deer abundance. Public trust stewardship of North American landscapes will require a fundamental overhaul in deer management to provide for a brighter future, and oak seedlings may be a promising metric to assess success. These changes will require intense public debate and may require new approaches such as regulated commercial hunting, natural dispersal, or intentional release of important deer predators (e.g., wolves and mountain lions). Such drastic changes in deer management will be highly controversial, and at present, likely difficult to implement in North America. However, the future of our forest ecosystems and their associated biodiversity will depend on evidence to guide change in landscape management and stewardship.

## Graphical abstract

We show that neither surgical sterilization nor recreational hunting can sufficiently reduce white‐tailed deer populations or their impacts on oak seedlings in eastern North America. Using trained archers and night‐time shooting over bait shows promise as a management tool. Fundamental changes in deer management are required to recover healthy and diverse forests.

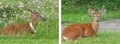

## 1 INTRODUCTION

**Figure 1:**
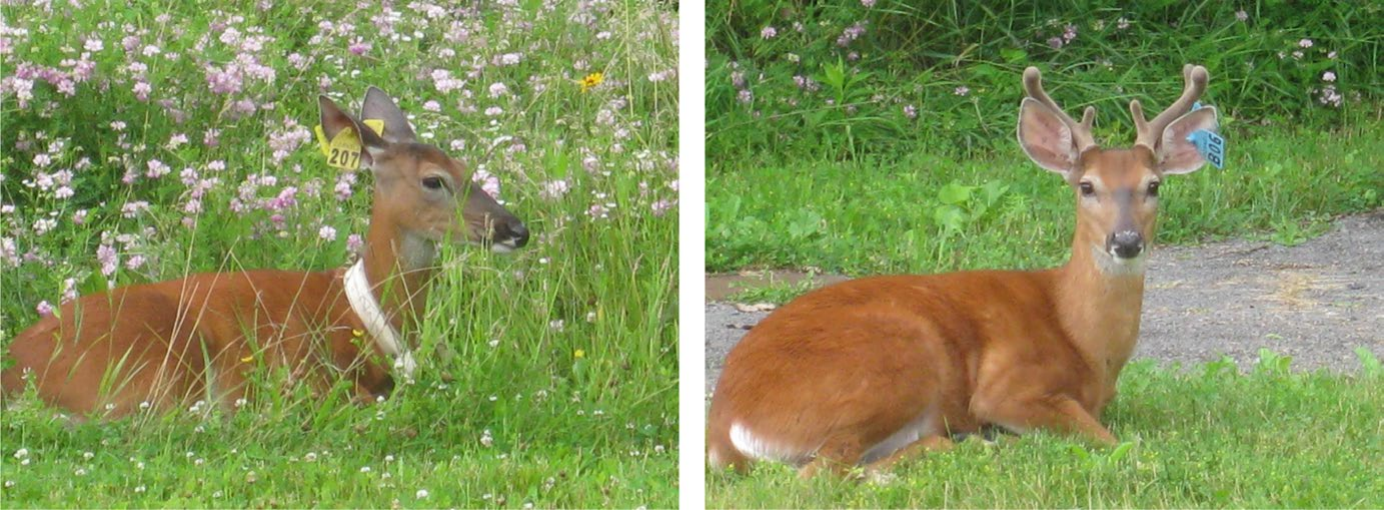
White‐tailed deer female (yellow ear tag and VHF collar) and male in velvet (blue ear tags) on the Cornell campus in summer 2009 (photos by B. Blossey)

Temperate forests in eastern North America face a crisis due to accelerated development, climate change, and introduced pests and diseases (Aukema et al., 2010; Liebhold et al., 2013). In addition, high populations of white‐tailed deer (*Odocoileus virginianus*, Figure 1) cause dramatic and wholesale changes in habitats across much of North America, that threaten the continent's biodiversity, economies, and human health (Côté, Rooney, Tremblay, Dussault, & Waller, 2004). This once iconic species has turned into an ecological villain and human health threat, yet modern societies struggle to find appropriate responses (Sterba, 2012).

Overexploitation nearly led to extinction of white‐tailed deer in the late 1800s. However with changes in hunting regulations and establishment of state wildlife agencies to manage recovery of the species in the early 1900s, deer herds rebounded quickly (Halls, 1984). Population recovery was aided by subsidies from human activities (agriculture) and the regrowth of eastern forests. Early dire warnings about long‐term ecological consequences of deer population increases in the absence of traditional predators, such as mountain lions (*Puma concolor*) and timber wolves (*Canis lupus*; Leopold, Sowls, & Spencer, 1947) were ignored by state wildlife agencies. Today, scientific evidence regarding negative impacts of historically high white‐tailed deer populations is voluminous, increasing, and largely uncontested.

White‐tailed deer are ruminant browsers with a variable diet composed of woody species, herbs, grasses, and mushrooms. Diet composition is influenced by geography, season, habitat features, primary human land uses, deer abundance, legacy effects, and plant community composition (Anthony & Smith, 1974; Arceo, Mandujano, Gallina, & Perez‐Jimenez, 2005; Daigle, Crete, Lesage, Ouellet, & Huot, 2004; Johnson et al., 1995; Nixon, Hansen, Brewer, & Chelsvig, 1991; Ramirez, Quintanilla, & Aranda, 1997; Royo, Kramer, Miller, Nibbelink, & Stout, 2017). Deer make daily feeding decisions based on their seasonal nutritional needs, individual preferences, nutritional value and defense chemistry of forage species, and presence/absence of predators (Berteaux, Crete, Huot, Maltais, & Ouellet, 1998; Cherry, Warren, & Conner, 2017; Hanley, 1997; Lavelle et al., 2015; Masse & Cote, 2009). Differences in nutritional value and palatability among plant species lead to distinct feeding preferences. Although deer can adapt as food quality declines due to selective removal of the most desirable species, resulting in smaller deer with reduced body size (Simard, Cote, Weladji, & Huot, 2008). Deer continue to seek out strongly preferred plant species, even if they occur at low densities, further increasing threats of local extinction for particularly vulnerable populations (Erickson et al., 2017).

Long‐term consequences of high deer populations have been documented for herbaceous and woody species alike. The impact of deer browse on herbaceous species may result in direct mortality, but tissue removal preventing flowering and reproduction has dramatic demographic consequences that play out on a decadal time scale. For example, high deer populations caused declines of >90% for many orchids in the mid‐Atlantic region in Maryland (Knapp & Wiegand, 2014). Deer browsing also threatens understory herbs like Trilliums (*Trillium grandiflorum* and *T. erectum*) and American ginseng (*Panax quinquefolius*; Bialic‐Murphy, Brouwer, & Kalisz, 2019; Dávalos, Nuzzo, & Blossey, 2014, 2015a; Knight, Caswell, & Kalisz, 2009; McGraw & Furedi, 2005), however, these are only a few well‐researched examples, and threats are widespread (Frerker, Sabo, & Waller, 2014). In contrast to herbaceous species that experience deer browsing without reprieve, most woody plants have the ability of vertical escape once terminal shoots grow out of browse height (1.5–2 m). However, current deer densities across much of eastern North America prevent transition from seedlings (<1 year old; up to 20 cm tall) to saplings (Kelly, 2019; Long, Brose, & Horsley, 2012; Miller & McGill, 2019). Despite abundant seed production by mature overstory trees and successful germination, deer browsing is now so extensive that forest regeneration after harvests or natural mortality is largely prevented, creating a regeneration debt (Miller & McGill, 2019) that plays out over centennial time scales and affects not just the highly palatable species. High deer browse pressure not only creates less diverse forests that will exist long into the future, but it also prevents dispersal of many tree species northward in response to climate change, which in turn has large economic consequences for timber management (Côté et al., 2004), and limits potential for climate change mitigation through reforestation (Bastin et al., 2019).

High deer populations and their impact on primary producer diversity and abundance led to dramatic abundance declines in forest macrolepidoptera specialized on understory plant species in New Jersey (Schweitzer, Garris, McBride, & Smith, 2014). In Pennsylvania, aboveground insect abundance, richness, and diversity were up to 50% higher where deer were excluded for 60 years (Chips et al., 2015). Furthermore, deer facilitate spread of invasive plants and invasive earthworms (Dávalos, Nuzzo, & Blossey, 2015b; Dávalos, Simpson, Nuzzo, & Blossey, 2015; Eschtruth & Battles, 2009; Kalisz, Spigler, & Horvitz, 2014; Shelton, Henning, Schultz, & Clay, 2014), which individually and collectively have far reaching consequences on soils, erosion, nutrient cycling, and food webs (Maerz, Nuzzo, & Blossey, 2009; Nuzzo, Maerz, & Blossey, 2009). In summary, elevated deer densities create depauperate landscapes, and the resulting successional forest trajectories have long‐lasting (>100 years) legacy effects that negatively affect all trophic levels including migratory birds (Bressette, Beck, & Beauchamp, 2012; Martin, Arcese, & Scheerder, 2011; Nuttle, Ristau, & Royo, 2014; Nuttle, Yerger, Stoleson, & Ristau, 2011). High deer populations also represent a human health threat due to deer‐vehicle collisions and amplification of tick populations and prevalence of tick‐borne diseases including Lyme (Kilpatrick, LaBonte, & Stafford, 2014; Raizman, Holland, & Shukle, 2013).

In the US, legal authority to manage deer and other wildlife as a public trust resource (except for endangered or migratory species) rests with state wildlife agencies, which follow the North American model of wildlife management, with hunting and trapping as core management tools (Geist, Mahoney, & Organ, 2001; Hare & Blossey, 2014; NYSDEC, 2011). However, the assertion that recreational hunting as currently implemented and regulated can achieve deer population regulation has been challenged (Williams, DeNicola, Almendinger, & Maddock, 2013). Further complications arise from strong opposition to hunting and lethal deer management by animal rights groups, particularly in suburbia (Sterba, 2012).

We used simultaneous experimental implementation of different deer management approaches (no management, sterilization, and recreational hunting) to assess competing claims by wildlife agencies (recreational hunting is able to control deer populations and their impacts) and animal rights activists (nonlethal control can reduce deer populations, and deer do not drive ecosystem deterioration). We know of no other study that simultaneously assessed effects of different deer management approaches for their effect on the size of a free‐roaming deer population and the impact on ecological resources. We used browse incidence and seedling growth of a bio‐indicator, red oak (*Quercus rubra*) to assess outcomes of different deer management approaches. The species is widespread in eastern North America, an important timber species, a major source of food for wildlife, and a species of intermediate preference for deer (Averill, Mortensen, Smithwick, & Post, 2016; McShea et al., 2007; Tallamy & Shropshire, 2009). In addition, *Q. rubra*, like other oak species, shows regional regeneration failures in eastern North America (Abrams & Johnson, 2012), but the species is flourishing when deer numbers are kept low, for example on tribal lands (Reo & Karl, 2010). We chose to focus on browse frequency and growth as the important variables determining the likelihood of seedlings to advance to the sapling stage in woody plant recruitment (Kelly, 2019). We included rodent attack, insect herbivory, and the role of competing vegetation into our assessments (a more complete justification for our approach is detailed in Section 2.3) due to their potential influence on oak recruitment and demography (Crow, 1988; Davis, Tyler, & Mahall, 2011). We evaluated the following hypotheses:

1. Deer browse intensity on red oak seedlings will vary in different management zones. Specifically, we expected browse rates to be highest in the no management zone, be intermediate in the sterilization zone, and be lowest in areas with recreational hunting.
2. The proportion of oak seedlings browsed by deer will be higher than the proportion of oaks affected by other factors (rodents, insects, and winter mortality).
3. Oaks protected from deer herbivory will grow, while height of oaks exposed to deer herbivory under the same forest conditions will regress or remain stable.
4. Browse intensity on red oak seedlings is a function of the deer population size.

## 2 MATERIALS AND METHODS

### 2.1 Study area and deer population estimation

**Figure 2:**
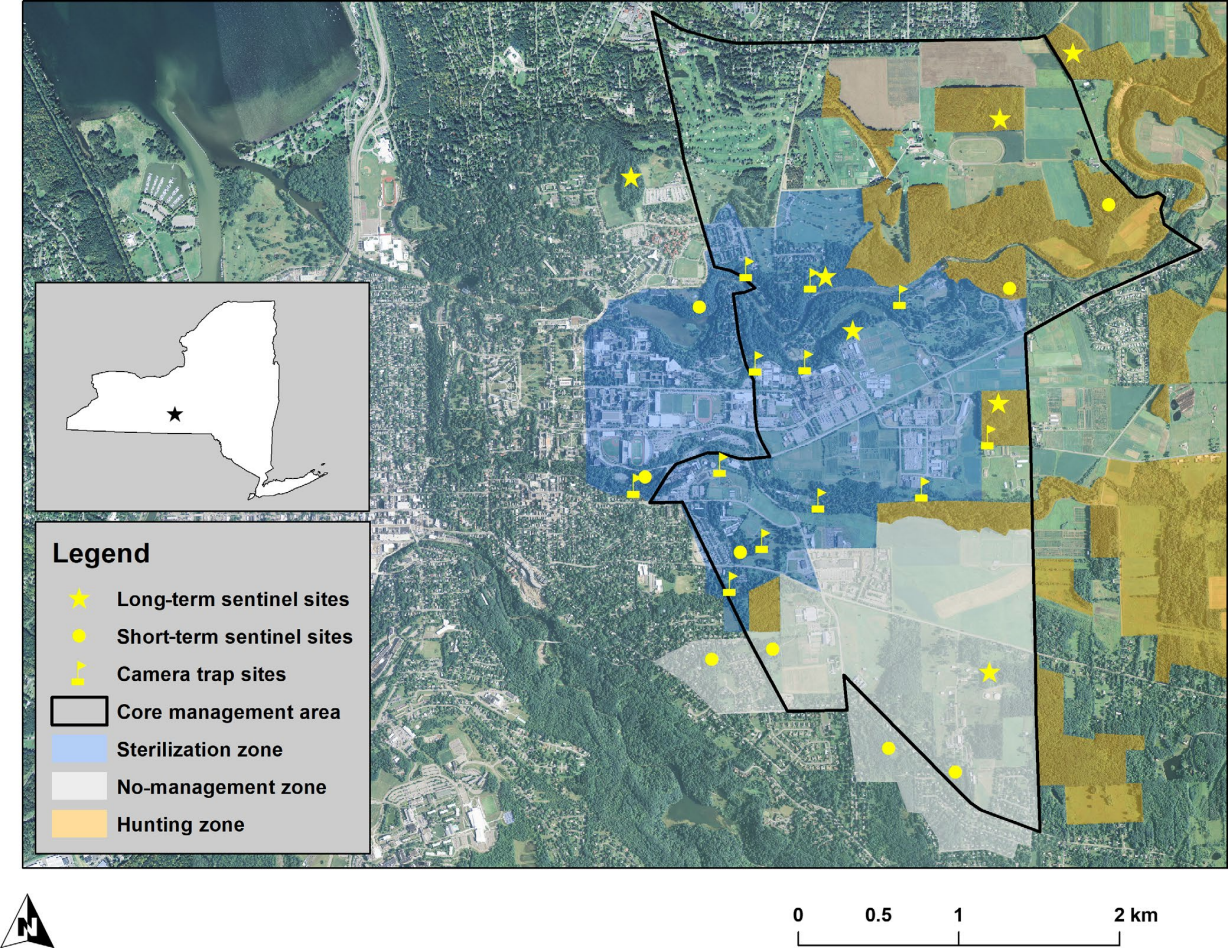
Delineation of no management, sterilization, and hunting zones (2008–2013) and core deer management area (after 2013) surrounding the main Cornell University campus in Ithaca, New York, USA. Short‐term (2010 and 2011) and long‐term (2010–2015) *Q. rubra* planting and camera trap locations are indicated by yellow markers

Our study area was located in central New York State, USA, and incorporated major portions of the Cornell University campus and surrounding areas in the Towns of Ithaca and Dryden (Figure 2). Historically, hunting, as regulated by the New York State Department of Environmental Conservation (NYSDEC), has occurred on Cornell University lands for decades. Lack of success in reducing deer populations and their associated impacts resulted in the establishment of an Integrated Deer Research and Management (IDRM) Program in 2007 (Boulanger, Curtis, & Blossey, 2014). The goal of this program was to reduce deer populations, human health threats, and ecological and economic deer impacts by 75% over a 10‐year time frame. Core elements of IDRM were coordination of deer management efforts, surgical sterilization, a recreational hunting program, monitoring of deer abundance on core campus, and assessment of ecological health using bio‐indicators.

We initially established three zones with different deer management approaches: (1) no management (approx. 281 ha) where neither sterilization nor hunting was permitted; (2) sterilization (approx. 446 ha); and (3) a hunting zone (approx. 1,600 ha) where recreational hunting (bows, crossbows, and firearms) occurred in accordance with local and state laws (Boulanger et al., 2014). These three zones did not overlap but were adjacent to each other, each representing a mix of suburban, residential and rural agricultural and forested lands (Figure 1).

Obtaining accurate estimates of abundance for free‐ranging deer is notoriously difficult and cost prohibitive, particularly over large areas. Traditional survey methods have included track or pellet counts, spotlight surveys, drive counts, aerial or thermal imagery surveys, or population reconstruction based on hunter reports and sex ratios. However, all of these methods produce unreliable results, and some may only be available in open habitats (Fritzen, Labisky, Easton, & Kilgo, 1995; Goode et al., 2014; Keever et al., 2017; Marques et al., 2001; Norton, Diefenbach, Wallingford, & Rosenberry, 2012). Lately, use of camera traps has become popular. However, accurate population estimation still requires identification of individuals, and individual deer are impossible to distinguish, except for branch‐antlered male deer (hereafter bucks) in the fall. Furthermore, density estimates are influenced by detection probabilities that vary seasonally and with terrain, human development, and hunting pressure (Parsons et al., 2017). The development of genetic tools using DNA extracted from pellet groups to estimate deer density and spatially explicit habitat use shows great promise (Brinkman, Person, Chapin, Smith, & Hundertmark, 2011), but costs associated with sample processing make this still cost prohibitive in most circumstances (Goode et al., 2014).

**Figure 3:**
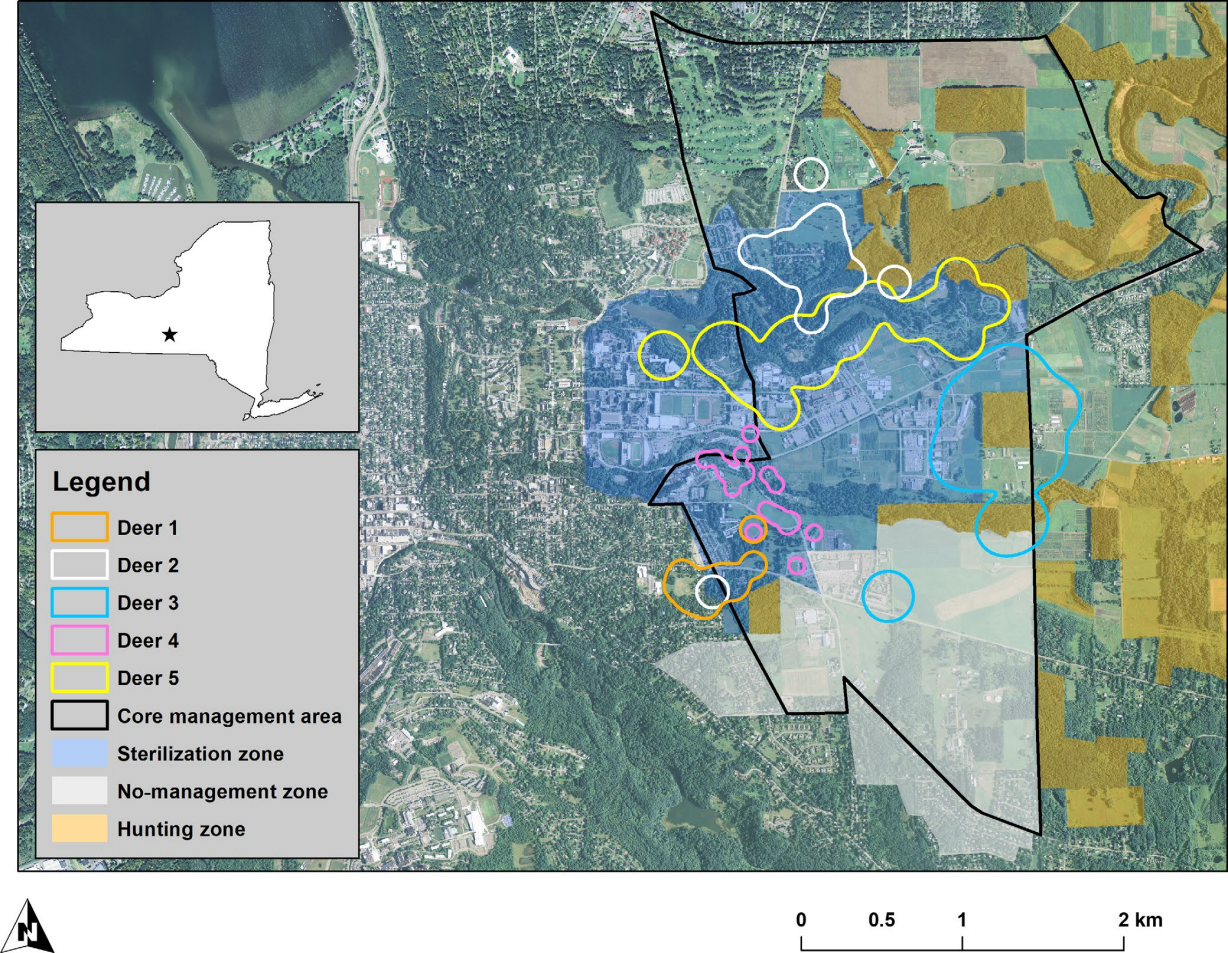
A sample of variation in shape and size of 95% adaptive kernel home range estimates for surgically sterilized radio‐collared adult female deer on Cornell campus (2008–2013; adapted from Boulanger et al., 2014)

To obtain accurate deer population estimates to quantify responses to our management activities, we utilized a cohort of 120 individually marked deer. We captured and sedated deer in the sterilization zone (Figure 2), and veterinary surgeons performed tubal ligations and ovariectomies (Boulanger & Curtis, 2016). We captured most of the 120 deer in the first two years of the program, but continued to target immigrating individuals to maintain a high sterilization rate. We fitted captured deer with individually numbered livestock ear tags (Premier1 Supplies) and fitted most sterilized adult females with very high‐frequency (VHF) radio collars (Telonics, Inc.; Figure 1). We released all deer at their original capture location and monitored their movements, which varied widely among individuals (Figure 3). We then conducted an annual camera census (mark‐recapture study) in the sterilization zone each spring using 12 digital infrared‐triggered cameras that took pictures at bait stations continuously for 5–7 days. Our population estimation thus occurred at a time when potential behavioral responses to fall hunting pressure and spatial escape of deer into the sterilization or no‐hunting zones would have been minimal. We placed cameras in a grid system comprised of 40‐ha blocks (Figure 1) and calibrated them to take a photograph every four minutes, if deer were present at bait. We tallied photographs and then modeled deer abundance using programs MARK and NOREMARK (Curtis, Boldgiv, Mattison, & Boulanger, 2009; White, 1996). An initial test of this approach obtained accurate and precise estimates of deer abundance (Curtis et al., 2009).

### 2.2 Deer management

In addition to continuing sterilization efforts of deer immigrating into our sterilization zone, we established a coordinated recreational hunting program in accordance with New York State hunting seasons each fall from October to December. For safety reasons, we restricted hunting close to campus or suburban neighborhoods to archery, but elsewhere allowed shotguns and/or muzzleloaders. We experimented with various approaches to increase antlerless harvests by the >500 recreational hunters who annually registered for the Cornell University Hunting Program. These included Earn‐A‐Buck approaches (hunters were required to shoot a female before they can shoot a buck), and use of Deer Management Assistant Permits (additional nonantlered tags) issued by the NYSDEC. Beginning with the 2012 season, the NYSDEC established a special Deer Management Focus Area that allowed harvest of two antlerless deer per hunter per day through the regular hunting season and added a unique 3‐week antlerless season in January that included our core management area (Boulanger et al., 2014) to assist in deer management efforts.

Despite hundreds of deer taken by hunters on Cornell lands and doe sterilization rates of >90%, our camera surveys indicated that by 2012, five years into the program, we had not achieved any reduction in the core deer population (Boulanger & Curtis, 2016). In response to our failure to reduce the deer population, we eliminated sterilization efforts and established a larger core management area (CMA, approx. 953 ha) that included most of the sterilization zone plus selected areas previously designated as no management or hunting zones (Figure 1). In 2013 and 2014, we allowed recreational archery hunting in designated areas of the CMA during the hunting seasons and added use of Deer Damage Permits (DDPs) as permitted by NYSDEC. Use of DDPs allowed use of bait (typically maize [*Zea mays*]) and shooting at night using artificial lights, both of which are otherwise illegal in New York State, from the end of the regular season in December to the end of March the following year. We allowed use of bows and crossbows with no tag limits placed on volunteer participants. Each participant was further required to report their efforts (hours in stand), the fate of every arrow shot, distance lethally wounded deer travelled, wounding rates, and other observations. This allowed us to make adjustments in the program as needed and be accountable to hunters, the state management agency, university administration as well as those questioning methods and security of our approach. In 2015, we eliminated all recreational hunting in our CMA and focused exclusively on volunteer archers using DDPs to limit behavioral changes in deer exposed to hunting pressure (Williams, DeNicola, & Ortega, 2008). Our highly structured DDP program restricts shooting at bait locations to no more than once per week (or less) in an attempt to limit deer behavioral changes while increasing our ability to achieve management goals. Recreational hunting has continued outside of the core management area. In addition, two adjacent villages (Cayuga Heights and the Village of Lansing) use their own DDPs to remove deer, while the City of Ithaca has a discharge ordinance that prohibits the ability to take deer within City limits.

### 2.3 Indicator selection, *Q. rubra* natural history, seedling performance, and procedures

Ideally, any comprehensive measurement of the status of forest biodiversity should include multiple metrics or indicators at different trophic levels; however, there are currently no agreed upon or sensitive metrics available. While desirable, it is typically impossible to measure many different variables in different trophic levels when assessing outcomes of human activities, including landscape or deer management, effects of pollution, etc. However, applied ecology has a long history of using indicator species (Bachand et al., 2014; Dale & Beyeler, 2001) to better gauge the outcome of management interventions. Using an indicator species, or a restricted portfolio of indicators, would also facilitate adoption of metrics by land managers who do not have the resources nor expertise that typically are required in scientific experiments. For the purpose of assessing differences in outcomes of alternative deer management approaches, an indicator should be sensitive to changes in deer browse pressure, for example due to fencing or culling.

We selected *Q. rubra* as our bio‐indicator to assess the impact of different deer management approaches or changes in deer abundance on ecological health. In a previous study (Blossey, Dávalos, & Nuzzo, 2017), we demonstrated the utility and sensitivity of *Q. rubra* to respond to changes in deer browse pressure (fencing) through improved growth. We chose *Q. rubra* for multiple reasons, including its potential to serve as a general indicator of forest health that can be planted with reasonable expertise at low cost. This allows communities or individual landowners to assess whether their selected deer management approaches result in improvements in the ability to regenerate a diverse forest that includes *Q. rubra*. Many different oaks, including *Q. rubra* have shown persistent regeneration failures in the Northeast for decades, and various factors including lack of fire, too much shade, and high deer browse pressure are implicated (Abrams, 2003; Abrams & Johnson, 2012). These regeneration failures, as in many other woody species, occur despite abundant mature oak trees that mast frequently followed by successful acorn germination. However, seedlings are unable to advance to the sapling stage, a pattern that can be reversed through fencing, suggesting that deer play an important role in preventing this transition (Abrams & Johnson, 2012; Leonardsson, Lof, & Gotmark, 2015; Long et al., 2012; Long, Pendergast, & Carson, 2007; Schwartz & Demchik, 2015; Thomas‐Van Gundy, Rentch, Adams, & Carson, 2014). These patterns suggested that selecting *Q. rubra* was an appropriate and sensitive indicator for assessing the outcome of our different deer management approaches. Changes in browse frequency for *Q. rubra*, while not expected to be identical for other species, should indicate the direction of overall browsing pressure experienced by other taxa.

*Quercus rubra* is a widely distributed deciduous tree in eastern North America ranging from Ontario and Quebec south to Georgia and Alabama in the east, and from Minnesota and Iowa south to eastern Oklahoma, with isolated populations in Louisiana (USDA NRCS, 2017). Mature trees are typically 20–30 m tall, start to produce acorns at age 30–40, and may live for up to 500 years. Wood of *Q. rubra* is widely used to make furniture, veneer, cabinets, and flooring. Due to its vibrant fall foliage and qualities as a shade tree, *Q. rubra* was widely planted as an ornamental. Acorns need 2 years to mature, require cold stratification after dropping off the tree, and all surviving acorns germinate in the following spring. There is no seed bank. Mass fruiting occurs every 2–5 years. Acorns may be consumed by insects, many mammals, and birds. Successful seedling recruitment is episodic and often only occurs after mass‐fruiting events due to insect attack and acorn predation, particularly by rodents (Crow, 1988). Depending on site conditions, young trees may need to spend many years, or even decades, in the forest understory before gap creation due to natural mortality or harvesting of overstory trees creates opportunities to enter the overstory.

For *Q. rubra*, germination and seedling establishment is possible on many different soils, and in full or partial shade. Seedling and sapling densities of 1,000–2,500 stems/ha are required to ensure sufficient regeneration for future canopy recruitment, and in many places in the Northeast sapling densities are much lower indicating a regeneration debt (Miller & McGill, 2019). Competing herbaceous vegetation, poor soils, or shade intolerance have been proposed as factors limiting the ability of *Q. rubra* to survive more than a few years in the understory (Abrams, 2003; Crow, 1988; Lorimer, Chapman, & Lambert, 1994). However, experimental investigations have shown that oak seedlings are similarly shade tolerant as many other species, (no growth or survival benefits beyond 15% full sun'; Dillaway, Stringer, & Rieske, 2011; Kaelke, Kruger, & Reich, 2001; Long et al., 2012). Liming does not affect oak seedling growth (Long et al., 2012), and fire and herbicide treatments to reduce effects of competing vegetation actually negatively affect oak seedlings compared with untreated controls (Miller, Brose, & Gottschalk, 2016). However, in all these studies, fencing had substantial and sustained beneficial effects on oak seedling growth and survival. SORTIE, a model to predict Northeastern hardwood forest successional dynamics based on field assessments, indicates that a 1‐cm‐diameter *Q. rubra* sapling has a 30% probability to survive for 5 years in 1% sunlight, and it will take 125 years to reach 3 m in height (compared with 12 years in full sun; Pacala et al., 1996). Unfortunately, SORTIE, as so many other early investigations into forest regeneration failures, ignores the transitions in the very early life history of *Q. rubra*. It also does not incorporate biotic pressures (insect, rodent, or deer browse intensity), which, as recent evidence suggests (Kelly, 2019; Miller & McGill, 2019), appear crucially important, but are also difficult to capture if deer rapidly consume emerging seedlings.

Matrix populations models (Caswell, 2001), while popular with ecologists for many different species, have not been used frequently for long‐lived species such as oaks, and none exists for *Q. rubra*. Therefore, we can only speculate about the importance of shade, other abiotic factors, competition, insect, rodent, or deer herbivory on the demography of *Q. rubra* and in prohibiting transition from germinated seedling to sapling. The successful transition from seedling to sapling and vigorous sapling growth in fenced plots suggests that deer browse is of overriding importance. This is supported by elegant experiments to assess the importance of fecundity and biotic factors (cattle, deer, and rodents) on population growth rates of Valley oak (*Quercus lobata*) in California (Davis et al., 2011). While survival rates for *Q. lobata* varied among years, population growth rates were primarily limited by survivorship and growth of established seedlings and saplings, which were strongly affected by ungulate browsing and rodent damage. The terminology and criteria distinguishing seedlings from saplings vary among investigators (typically height or stem diameter). In our assessment, we follow natural history and, in part, the demographic model using *Q. lobata* (Davis et al., 2011). We define seedlings as oaks that recently germinated and are <20 cm tall. We define saplings as individuals >20 cm tall, regardless of age.

We were not interested in building a full demographic model, but we were looking for a quick assessment (every year or in short intervals) that allowed us to evaluate whether differences in deer management approaches and changes in deer abundance would affect the growth and transition from seedling to sapling for *Q. rubra*. We therefore chose to assess deer browse frequency and rodent or insect attack in annual oak cohorts that we followed for a growing season up to a year. We incorporated rodent and insect attack into our assessments due their importance in affecting oak seedling survival and growth in other studies. We did not focus on survival, because browsed oaks, or oaks cut by rodents may produce secondary sprouts with very small leaves, and these individuals may linger for many years (very few return to vigorous growth; B. Blossey personal observation). We also chose to plant propagated oaks to standardize our approach across many different forests. In many of our local forest fragments, naturally germinating oak seedlings are extremely rare, occur only in microsites protected from deer browse, such as in treefalls or on steep slopes, are not produced annually, and their abundance varies with overstory tree composition. This variation prevented use of naturally occurring *Q. rubra* seedlings for our assessments.

Each September and October, we collected *Q. rubra* acorns from local sources and stored them over winter in gauze bags buried in moist sand in a dark walk‐in environmental room (Nor‐lake) at 4°C. We planted acorns each February/March in individual SC7U Ray‐Leach Cone‐tainers (3.8 cm diameter × 14 cm deep; Stuewe and Sons) using commercial potting soil (Farfard Canadian growing mix No. 1‐P) and allowed them to germinate and grow in a greenhouse (20–25°C daytime, 10°C at night) under natural photoperiod. After seedlings developed 2–4 leaves (late April to mid‐May), we hardened them outside on elevated metal greenhouse benches with legs standing in buckets filled with soapy water to prevent earthworm colonization. We protected seedlings against deer or rodent herbivory in walk‐in field cages (Lumite^®^ screening, shade 15%, porosity 1629CFM; Synthetic Industries).

**Figure 4:**
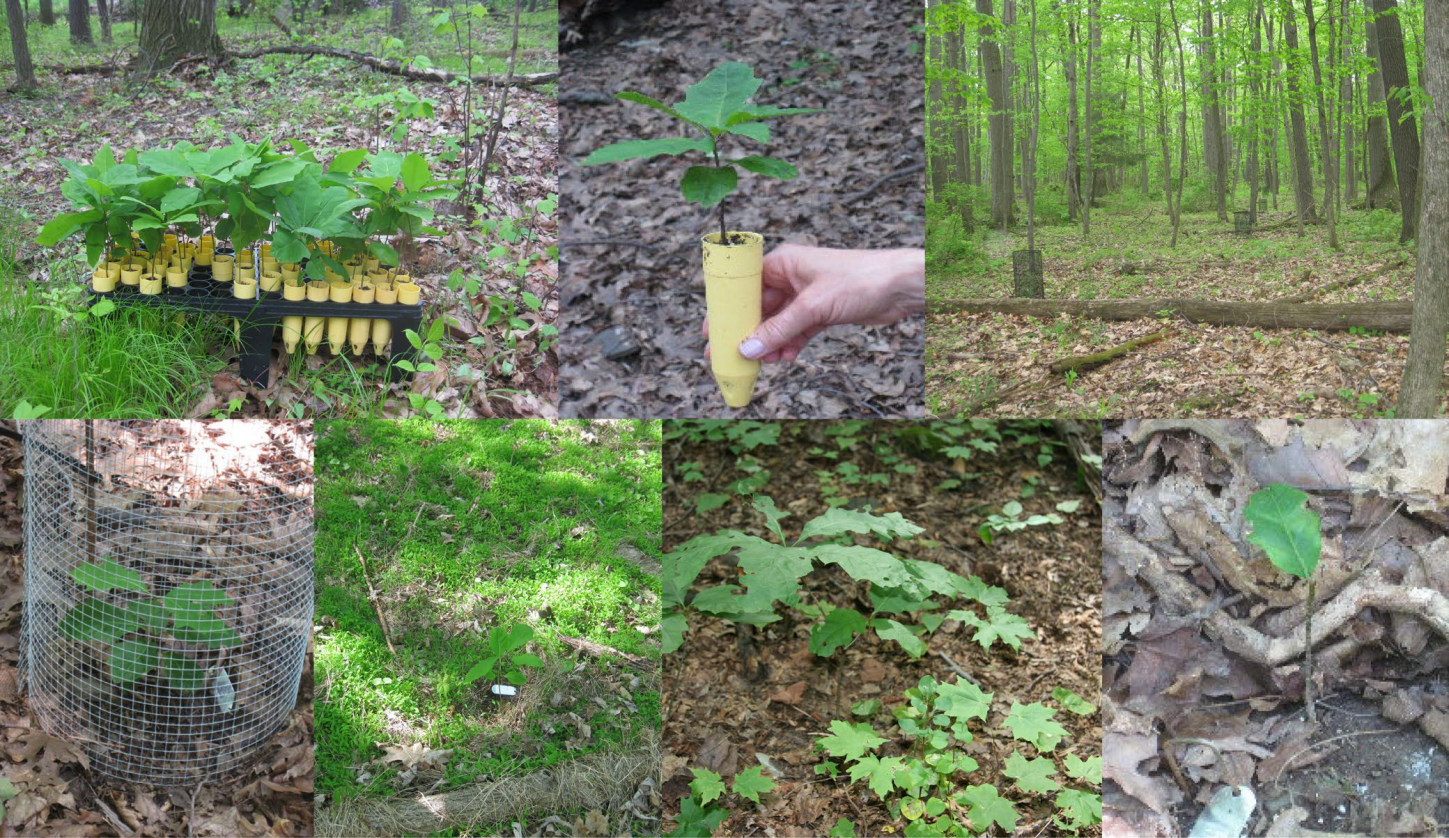
Top row L to R: Oaks seedlings ready to transplant, individual oak, and field cages to protect seedlings. Bottom row L to R: Healthy oak protected by wire‐mesh cage, oak in matrix vegetation, healthy surviving oak, and partially browsed oak with a single leaf remaining (all photos by B. Blossey)

For each site, we selected 40 well‐watered seedlings with 3–8 leaves (Figure 4) usually 8–15 cm tall. We typically selected a 100 m × 100 m area and planted seedlings >3 m apart along multiple meandering transects (Figure 4) from mid‐May to mid‐June, the same time field germinated oaks would appear in our region. We avoided planting seedlings next to live large trees or in windfalls, on very steep slopes, or among large boulders that could function as refuges by limiting physical access by deer. We used a handheld drill with a 5‐cm diameter, 30‐cm long masonry drill bit to create tapered planting holes (10–15 cm deep × 5–10 cm wide). We removed rooted seedlings from their Cone‐tainers, removed the acorn (to reduce rodent predation), and then planted seedlings firmly covering potting soil with local soil. We placed a numbered metal tag (Racetrack aluminum tags; Forestry Suppliers) staked into the ground next to each seedling. Immediately after planting, we measured seedling height (cm), recorded the number of leaves, and then measured “average” height of vegetation at four locations approximately 50 cm away from the seedling (for seedlings planted in 2010 only). Surrounding vegetation could either function as aboveground competition, or possibly as camouflage, and hence protect oak seedlings (Underwood, Inouye, & Hambäck, 2014). We protected half of the seedlings at each site (randomly alternating caged and uncaged oaks) with individual wire‐mesh or plastic hardware net cages (Tenax Corporation; 50 cm diameter × 1 m tall, mesh size 1 × 1 cm, Figure 4), to prevent deer access.

We revisited each planting location after 7–10 days to assess each seedling (we recorded no transplant mortality), and thereafter at monthly intervals to record deer browse, rodent attack (recognized by a 45° cut angle), other herbivory or other causes of mortality (usually winterkill). We terminated monthly visits with leaf senescence in October and recorded attack one last time after leaf out in May or June 2011. We repeated the same procedures in 2011, using a new cohort of seedlings planted into the same locations. However, because most damage occurred before leaf senescence, we followed the 2011 cohort only until October. We lost one location in the no management zone; thus, we planted 600 oak seedlings in 2010 and 560 in 2011.

The assessments of the 2010 and 2011 cohorts allowed us to evaluate the impacts of no management (no deer removal, except through deer‐vehicle accidents), sterilization, and recreational hunting (Figure 1) on oak browse rates, rodent attack, and growth for oaks protected in individual cages or exposed to deer. Because our different management approaches did not result in sufficient deer population reductions, we changed our management regime beginning with the fall 2013 season (see Section 2.2 for details). We continued assessment of oak seedling browse and growth at a subset of seven sites located within or at the perimeter of the CMA (Figure 2) to assess whether deer browse rates on oak seedlings were sensitive to changes in the deer population from 2010–2011 to 2014–2015 (omitting 2012 and 2013 due to lack of funding). For the latter cohorts, we did not cage any oaks and therefore were able to reduce the number of planted oaks/site to 20. We continued to use baited camera traps to assess the status of the spring deer population each year and to determine whether our changes in deer management in the CMA resulted in herd reduction. Both camera trapping and oak sentinel assessments occurred at a time when known behavioral responses to fall hunting pressure and spatial escape of deer into areas without hunting pressure did not exist.

### 2.4 Data analysis

We evaluated deer browse rate as a function of management regime and fencing (open or caged) with Cox proportional hazard models implemented in the R statistical (R Core Team, 2016) package “coxme” (Therneau, 2015). We included initial oak height at planting and average vegetation height (for 2010 only) as covariates. We included site as a random factor in all models to reflect the hierarchical structure of the data. The test compared time (number of days since planting) to deer browse among experimental groups. Data were right‐censored because no information about oak browse rates was available after the study period. Deer browsed 113 oaks protected in cages (94 in 2010 and 19 in 2011) by physically dislocating fencing material to gain access. We excluded these oaks from further analyses after deer damaged fences. We used competing risk analysis package “cmprsk”, (Gray, 2014) to evaluate probability of an event (defined as a change in the status of an oak due to deer browse) occurring in the presence of competing factors (rodent attack and unknown mortality; Scrucca, Santucci, & Aversa, 2010). We excluded fenced oaks in Cox proportional models and cumulative risk analyses. We fitted separate models for oaks planted in 2010 and 2011 because we lost one study site in 2011.

We used linear mixed models (LMM, package lme4; (Bates, Maechler, Bolker, & Walker, 2014)) to evaluate effect of year, fencing, deer management regime, and second‐order interactions on daily growth rates (cm/day) of *Q. rubra* seedlings. We estimated growth rate as the difference in oak height between the first and last sampling date divided by the number of days between samplings. We included site as a random factor to reflect the hierarchical structure of the data. We used variance inflation factors (VIF) to assess collinearity among explanatory variables (Zuur, 2009). Variables were not correlated (VIF < 3).

We used generalized linear mixed models (GLMER) to evaluate the effects of management regime, fencing, and initial oak height on the probability of transitioning into a sapling stage. We used log‐likelihood tests between a full model and a model where we deleted the term of interest to assess significance.

We used Akaike Information Criterion (AICc; Burnham & Anderson, 2002) to evaluate explanatory power among competing models (for LMM, GLMER, Cox proportional hazard models, and competing risk analysis). We ranked candidate models according to the difference between model's AICc and min AICc (ΔAICc). We considered all models within two AICc to be similar. For LMM only, we evaluated percent variance explained by the model with conditional (full model) and marginal (fixed effects only) *R* ^2^ (Nakagawa & Schielzeth, 2013).

We used linear regression to evaluate changes in the proportion of oaks browsed during the growing season (June–October) as a function of spring deer abundance estimates. We calculated mean oak browse rate during the growing season per year across seven sites located within the core management area (Figure 1). Oak browse by site was estimated as the number of browsed oaks 200 days after planting over the total number of oaks planted at the site (*N* = 20).

## 3 RESULTS

**Figure 5:**
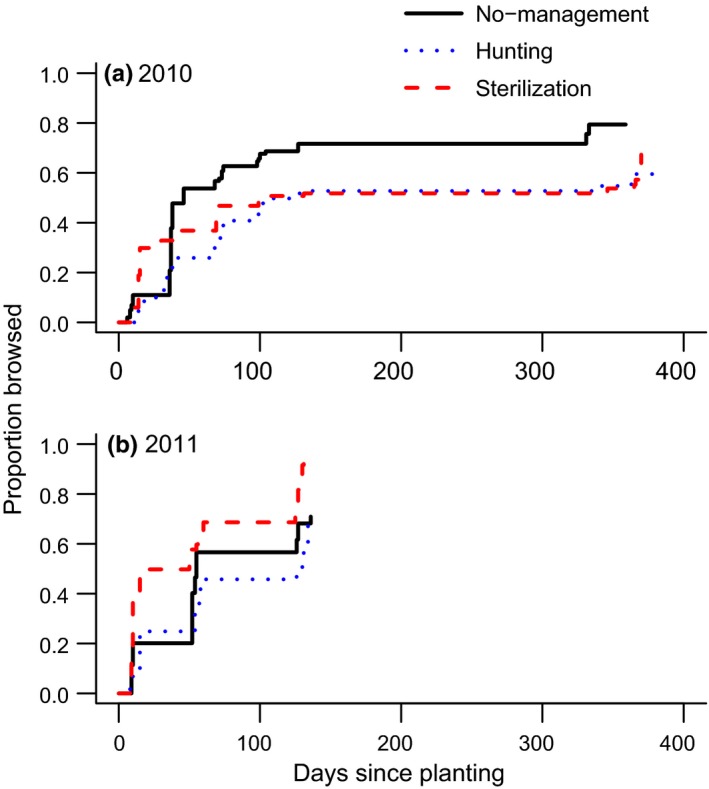
Proportion of browsed *Q. rubra* seedling cohorts planted in spring 2010 and 2011 in areas using different deer management (no management, hunting, or sterilization). Only unfenced oaks were included in the analysis (*N* = 20 oaks per site; 5 sites per management regime; one site in the no management area was omitted in 2011). Lines represent expected values according to mixed effects Cox regression (site included as random factor, Table 2). For clarity, we omitted standard errors

**Figure 6:**
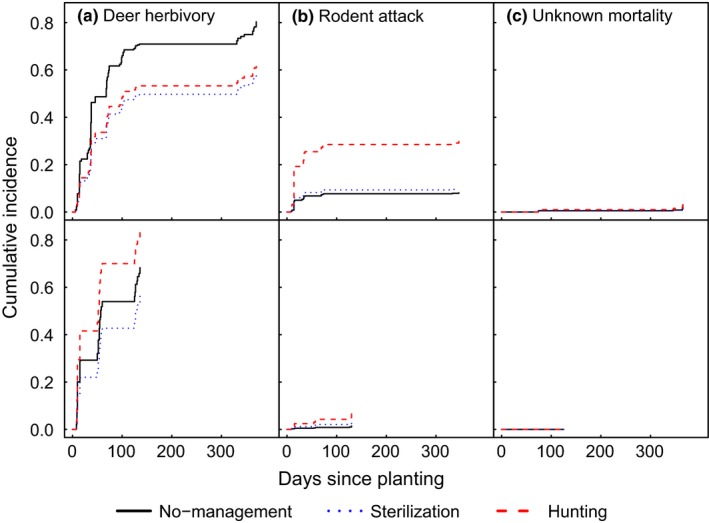
Cumulative incidence of deer herbivory (a), rodent attack (b), and unknown mortality (c) for unprotected *Q. rubra* seedling cohorts planted in spring 2010 (top row) and 2011 (bottom row) in areas with different deer management (no management, hunting, or sterilization; *N* = 20 oaks per site; 5 sites per management regime; one site in the no management area was omitted in 2011)

We encountered differences in the fate of *Q. rubra* seedlings among locations, management regimes, and in 2010 or 2011 cohorts (Table 1). Across all three management zones, deer browsed 65% of unprotected oaks (*N* = 196 of 300 planted in 2010 and 182 of 280 planted in 2011). In both years, but particularly in 2010, deer compromised and physically dislocated cages to gain access to protected *Q. rubra* seedlings (Table 1). Deer browse resulted in complete or partial removal of leaves, but most often deer removed entire upper stem portions of the seedling (Figure 4). Deer browse did not always result in immediate death, and surviving seedlings produced small replacement leaves. This also sometimes occurred after rodent attack that severed the stem a few cm above ground. Rodent attack and mortality due to unknown causes were similar for unprotected and fenced *Q. rubra* seedlings, but differed among deer management regimes and sites (Table 1). Deer browse and rodent attack occurred rapidly after planting, typically within 1–2 months before trailing off (Figures 5 and 6).

**Table 1:** Number of oaks browsed by deer, attacked by rodents, or dead due to unknown causes when planted without (open) or with individual mesh cages (fenced) in 2010 (15 sites, *N* = 600) and 2011 (14 sites, *N* = 560) at sites with different deer management regimes

Management	Deer		Rodent		Unknown mortality	
	Open	Fenced^a^	Open	Fenced	Open	Fenced
2010						
No management	79	35	12	8	2	10
Sterilization	58	29	32	29	4	9
Hunting	59	30	12	4	2	2
2011						
No management^b^	53	2	1	2	0	1
Sterilization	77	11	6	14	1	1
Hunting	52	6	3	1	0	0

a Deer browsed some oaks after breaching fencing. We excluded these oaks from analyses after fence breaches.

b One no management site was excluded in 2011.

In 2010, the risk of browsing by deer was significantly higher for *Q. rubra* seedlings in the no management zone compared with seedlings in hunting and sterilization zones (Figure 5; Tables 2A and S1A). The best model indicated that browse risk significantly increased as a function of initial oak height (Table S2A) and was associated with a significant interaction between management zone and initial oak height, such that taller oaks were more likely to be browsed in the no management zone than in the hunting and sterilization zones. In 2010, initial oak height at planting averaged 14.7 ± 0.13 cm and oaks in the sterilization zone were slightly but significantly shorter at planting (mean ± *SEM*: 13.88 ± 0.19 cm) than oaks planted in no management (14.99 ± 0.22 cm) or hunting (15.17 ± 0.24 cm) zones (*F*_2,594_ = 10.4, *p* < .005; a posteriori Tukey test *p* < .05). However, oak height at planting was similar between caged (14.5 ± 0.18 cm) and unprotected individuals (14.85 ± 0.17 cm; *F*_1,594_ = 2.01, *p* = .15) in each management zone. Average height of the surrounding vegetation at planting (measured only in 2010) was significantly lower in the sterilization zone (mean ± *SEM*: 6.9 ± 1.5 cm) than no management (15.1 ± 1.9 cm) and hunting (11.3 ± 2.7 cm) zones, but did not differ between hunting and no management zones (a posteriori Tukey test; *p* < .05). Average vegetation height at planting was not a significant variable in our analyses and dropped from the best model (Table S1A).

**Table 2:** Results for mixed effects Cox regression evaluating effects of fencing (fenced or open), deer management (no management, sterilization, and hunting,), and average vegetation height on oaks planted in 2010 (15 sites) and 2011 (14 sites)

	Coef (*SE*)	Exp (coef)	*z*‐Value	*p*
**(A) 2010**				
Fixed effects				
Management (hunting)	−0.14 (0.93)	1.15	0.15	.88
Management (sterilization)	2.30 (1.51)	9.89	2.18	.03
Initial height	0.09 (0.04)	1.10	2.34	.02
Initial height: management (hunting)	−0.05 (0.06)	0.85	−0.91	.36
Initial height: management (sterilization)	−0.20 (0.07)	0.82	−2.74	.01
Random effects	Std dev			
Site	0.27			
**(B) 2011**				
Fixed effects				
Management (hunting)	−0.37 (0.37)	0.70	−1.00	.32
Management (sterilization)	0.55 (0.36)	1.73	1.52	.13
Random effects	Std dev			
Site	0.46			

Note

We present only results for the best model. Estimates and standard errors (*SE*) reported from the model fitted with restricted maximum likelihood.

In 2011, we found a marginally significant effect of management zone (log‐likelihood test between the model including management zone and the null model: χ^2^ = 5.9, *df* = 2, and *p* = .05) and no significant effect of initial oak height at planting (log‐likelihood test between the model including height and the null model: *χ* ^2^ = 0.35, *df* = 1, and *p* = .85) on the risk of being browsed by deer. However, the best model (lowest AICc) included management zone (Table S1B) and indicated that the risk of deer browsing was highest in the sterilization zone, followed by the no management zone, and the hunting zone (Figure 5b; Table 2B). Initial height of oaks planted in 2011 averaged 12.9 ± 0.11 cm and did not differ among management regimes or fencing treatments (*p* > .05).

Cumulative risk analysis indicated that risk of deer herbivory was significantly higher than risk of attack by rodents or unknown mortality (Figure 6; Table 3). For oaks planted in 2010, the risk of deer herbivory was significantly higher in the no management zone than in sterilization or hunting zones, whereas risk of rodent attack was higher in sterilization than no management or hunting zones (Figure 6; Tables 3 and S2). Unknown mortality (almost exclusively winterkill) was similar across all management zones and significantly lower than the risk of being browsed by deer or attacked by rodents (Figure 6; Table S2). For oaks planted in 2011, risk of deer herbivory was significantly higher in the sterilization zone, but risk did not differ between no management and hunting zones (Figure 6; Table S2). Rodent attack and unknown mortality were similar across management zones and insignificant (Figure 6).

**Table 3:** Results of cumulative risk analyses evaluating effects of deer management (no management, hunting, and sterilization) and average vegetation height (cm) on risk of deer herbivory and rodent attack occurring in presence of competing factors for oaks planted in 2010 (15 sites) and 2011 (14 sites)

	Coef (*SE*)	Exp (coef)	*z*‐Value	*p*
**(A) 2010**				
Deer herbivory				
Hunting	−0.59 (0.16)	0.56	−3.68	<.001
Sterilization	−0.49 (0.18)	0.62	−2.75	.006
Rodent attack				
Hunting	0.20 (0.47)	1.22	0.42	.67
Sterilization	1.43 (0.40)	4.18	3.62	<.001
**(B) 2011**				
Deer herbivory				
Hunting	−0.33 (0.18)	0.72	−1.81	.07
Sterilization	0.44 (0.17)	1.55	2.56	.01

Note

Initial vegetation height was not significant and dropped from best models. The null model was the best model predicting unknown mortality (for 2010 and 2011) and rodent attack (2011). For procedures of model selection, see Table S2.

**Figure 7:**
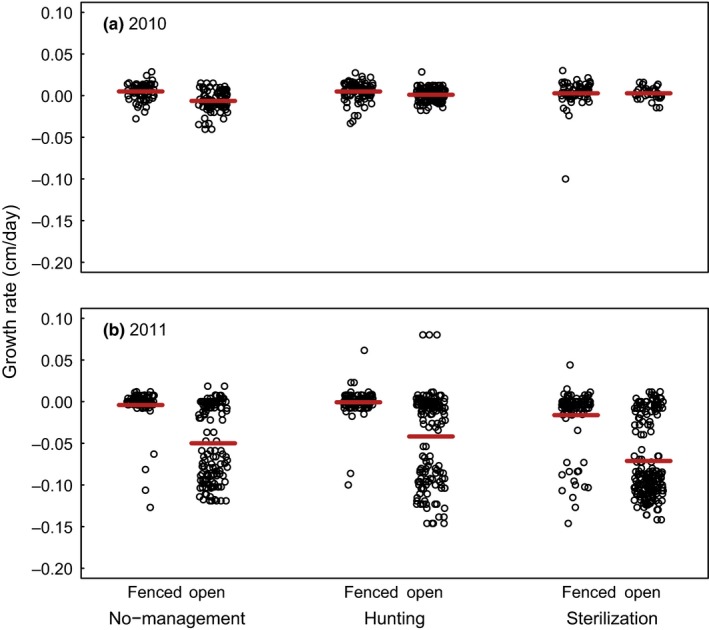
Growth (cm/day) of *Q. rubra* seedling cohorts planted in spring (a) 2010 and (b) 2011 at sites with different deer management (no management, hunting, or sterilization; *N* = 5 sites/management regime, one site omitted in the no management area in 2011). Oaks were either protected from deer in individual cages (fenced, Figure 4) or accessible by deer (open). Points (slightly jittered to reduce overlap) represent growth rates of individual seedlings and red horizontal lines indicate mean growth rate of caged and unprotected oaks in each management regime. For model results, see Table 4

Protected *Q. rubra* seedling grew significantly faster than unprotected oaks across all management zones in 2011 but not in 2010 (significant treatment × year interaction; Table 4; Figure 7). We also found a significant interaction between management regime and year (Table 4) such that growth rate was lower in the sterilization zone in 2011 compared with 2010 (Table 4). The proportion of variance explained by the fixed factors marginal *R* ^2^ = 0.40, whereas the conditional *R* ^2^ = 0.43, indicating the proportion of variance explained by the full model. Over the study period, 67 oaks transitioned into saplings (>20 cm; 64 and 3 of the 2010 and 2011 cohorts, respectively). Of the 67 oaks that transitioned into saplings, 54 were not browsed by deer, and 13 were browsed at least once. Probability of transitioning into saplings was significantly higher for unbrowsed oaks (*χ* ^2^ = 6.4, *df* = 1, *p* = .01) and positively correlated with initial planting height (log‐likelihood ratio; *χ* ^2^ = 234.36, *df* = 1, *p* < .001). Deer management zone had no significant effect on probability of transitioning into a sapling stage.

**Table 4:** Results of linear mixed model to evaluate effects of fencing, deer management regime (MR) and year planted on growth rate (cm/day) of fenced and deer accessible oak seedlings at 15 sites in 2010 and 14 sites in 2011

	Est	*SE*	*df*	*t*‐Value	*p*
Factor					
Intercept	0.002	0.004	40.23	0.36	.72
Year planted	−0.005	0.004	1,153.05	−1.17	.24
Treatment (open)	−0.006	0.003	1,165.00	−1.96	.05
MR (hunting)	0.004	0.006	25.57	0.72	.48
MR (sterilization)	0.001	0.006	37.64	0.18	.86
Year planted:Treatment (open)	−0.041	0.004	1,163.04	−10.19	.00
Year planted:MR (hunting)	0.003	0.005	1,163.75	0.55	.59
Year planted:MR (sterilization)	−0.019	0.005	1,164.31	−3.50	.00
Random effects	Std dev				
Site	0.007				

Note

Only results for the best model are presented. Estimates and standard errors are reported from the model fitted with restricted maximum likelihood. *p*‐Values are estimated using Satterthwaite's or Kenward–Roger's methods for degrees of freedom and *t*‐statistics (Kuznetsova, Brockhoff, & Christensen, 2017).

**Figure 8:**
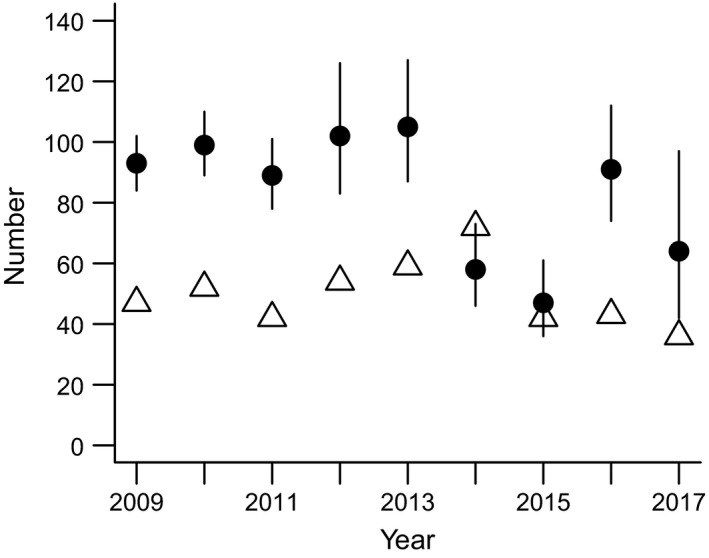
Annual spring deer population estimate (and 95%CI; circles; estimated using 12 infrared‐triggered cameras set over bait for 5–7 days) and number of deer removed the following fall/winter by volunteer hunters and deer‐vehicle accidents (open triangles) in the core management area (Figure 2). In some years, deer removals exceed spring population estimates due to immigration, rutting, or foraging activity typical in open ungulate populations

Our spring deer population estimates indicated a stable population in our CMA from 2009–2012 (Figure 7). With our switch to using DDPs in 2013, our 2014 spring population estimate for the first time indicated a reduced deer population and this trend continued in 2015, although immigration offset these gains in 2016 (Figure 8).

**Figure 9:**
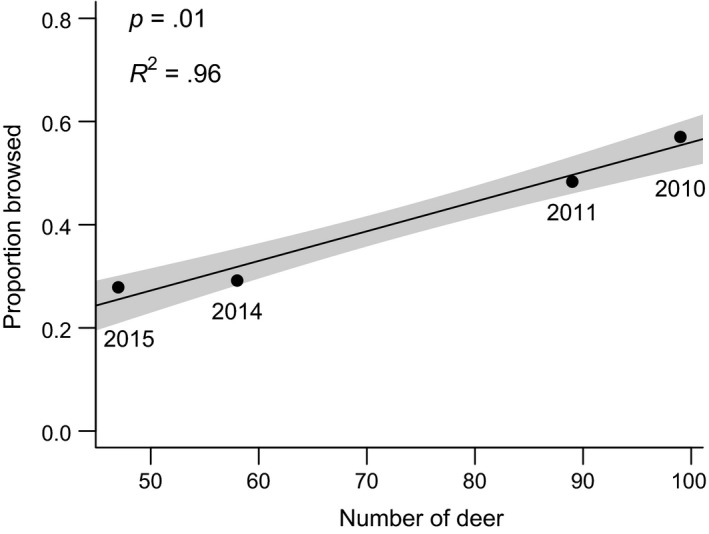
Proportion of *Q. rubra* seedlings browsed during the growing season (June–October) as a function of annual spring deer abundance (estimated using 12 baited infrared‐triggered cameras) in the core management area (Figure 2). Line and shaded areas depict linear model predictions and 95% CI

Annually, our hunters (and vehicle collisions) removed 40%–100% of the estimated spring deer population (a total of >440 deer from 2009 to 2017) from the CMA. Immigration, rutting activity, and foraging deer from areas adjacent to the CMA are included in this tally and indicate the importance of dispersal in open populations. Mean oak browse rate was significantly and positively correlated with mean deer spring abundance estimates (*F*_1,2_ = 71.5, *p* = .01; *R* ^2^ = 0.96; Figure 9); that is, as the deer population in the CMA was reduced, oak browse rates declined linearly. The proportion of *Q. rubra* browsed by deer varied annually and among the seven sites located within the CMA (Table S3).

## 4 DISCUSSION

Despite differences among locations and years, our study demonstrated that deer browse was the overwhelming threat to growth of unprotected *Q. rubra* seedlings, with rodents and other factors relatively unimportant (Figure 6), confirming our second hypothesis. These results align well with results of regional studies (Kelly, 2019; Miller & McGill, 2019) and the demographic model for *Q. lobata* in California (Davis et al., 2011), all indicating that after successful germination, seedlings are unable grow and transition to larger saplings under high deer browse pressure. This browse (and rodent attack) occurred rapidly in spring and early summer, and we would expect the same to occur for naturally germinating oaks. This will not allow seedlings to accumulate sufficient resources for successful regrowth should they be browsed, ultimately resulting in recruitment failure. In addition, because it occurs so rapidly after germination, and browsed seedlings are almost impossible to detect, even experienced observers will likely miss the deer browse effect on small seedlings.

We need to reject our first hypothesis. Differences in management regimes (no management, sterilization, or recreational hunting) did not result in meaningful differences in *Q. rubra* browse rates (Figure 5) despite some inconsistencies across years. This may not be surprising, given that we were initially unable to reduce the deer population in the CMA (Figure 8). There was a small but noticeably higher level of deer browse in the no management zone in 2010, but no differences in browse intensity among management regimes during 2011 (Figures 5 and 6).

Specifically, recreational hunting was unable to decrease deer densities sufficiently to protect growth of the majority of *Q. rubra* seedlings, as reported elsewhere (Bengsen & Sparkes, 2016; Blossey et al., 2017; Simard, Dussault, Huot, & Cote, 2013; Williams et al., 2013). This inability of woody species to transition from seedlings to saplings over much of the eastern US, and not just of palatable species (Kelly, 2019; Miller & McGill, 2019), occurs in a region where recreational hunting is widespread, ubiquitous, and accepted by the vast majority of citizens (Brown, Decker, & Kelley, 1984; Decker, Stedman, Larson, & Siemer, 2015). Some authors claim that hunting can reduce deer browse pressure on herbaceous and woody species, but browse reductions were either small (Hothorn & Müller, 2010), or we lack information about differences in hunting pressure in reference areas that also saw improvements in woody and herbaceous plant performance (Jenkins, Jenkins, Webster, Zollner, & Shields, 2014; Jenkins, Murray, Jenkins, & Webster, 2015). We therefore need to reject claims by wildlife management agencies that recreational hunting is sufficient to allow forest regeneration and can protect biodiversity (NYSDEC, 2011; Rogerson, 2010).

Animal rights and animal welfare organizations have long claimed that deer are not responsible for lack of forest regeneration and that there are more humane methods for managing populations (HSUS, 2018a, 2018b; PETA, 2018). However, there is no evidence to date that can support claims that fertility control alone can sufficiently reduce deer abundance in free‐ranging populations (Hobbs & Hinds, 2018; Raiho, Hooten, Bates, & Hobbs, 2015; Ransom, Powers, Hobbs, & Baker, 2014), including our own (Boulanger & Curtis, 2016). Examples cited as success stories show reduced fertility on islands or in fenced populations (Naugle, Rutberg, Underwood, Turner, & Liu, 2002; Rutberg, Naugle, Thiele, & Liu, 2004). To the best of our knowledge, no study has linked fertility control efforts to changes in other ecological parameters, such as changes in plant growth or plant communities, a long overlooked aspect of fertility control research (Ransom et al., 2014). Our study is the first attempt to associate performance of an indicator plant species to deer fertility control. We saw no evidence that fertility control is a viable tool for reducing herbivore populations or browse rates on *Q. rubra* seedlings in a fragmented suburban landscape. Despite a >90% doe sterilization rate and near elimination of deer fawns in our sterilization zone, the deer population remained stable due to immigration, particularly of bucks (Boulanger & Curtis, 2016). There was no reduction in the browse intensity on oak seedlings (Figures 5 and 6). Our results, including that oak seedlings protected from deer browse performed well at all sites, and results of other studies showing recruitment success in fenced areas, indicate that deer are indeed the major stressors in preventing forest regeneration. Our data offer no support for the promise of fertility control as a means to reduce deer browsing pressure.

We found support for our third hypothesis, that growing conditions at all our field sites enabled oak seedling growth (if protected by cages; unless compromised by deer; Figure 7), regardless of site‐specific growing conditions, differences in land‐use history, or potential presence of other associated stressors (invasive earthworms and invasive plants). Thus, at least in our area and probably across much of the eastern US, *Q. rubra* should be able to transition from seedlings to saplings successfully once white‐tailed deer populations are sufficiently reduced. We can also confirm our fourth hypothesis that the browse intensity on *Q. rubra* seedlings is a function of the deer population size (Figure 9), indicating that our sentinel approach is a sensitive and useful way to measure deer browse pressure and the success, or lack thereof, of different deer management approaches. We eventually achieved a deer population reduction (Figure 8) using methods typically not available to the recreational hunter, such as shooting over bait, and at night over extended periods. However, these intensive efforts will need to continue due to immigration pressure from the areas surrounding our CMA.

We are working with communities surrounding the Cornell campus to develop a regional approach. We are hopeful, although not certain, that collectively we may reduce deer populations to levels where *Q. rubra* seedlings will grow and ultimately transition to the sapling stage. Hunting, despite allowing access to every possible safe location on and near campus, removed about 50% (together with car accidents) of our annually estimated spring deer population in the CMA, and this temporary population reduction was not sufficient to affect oak browse rates or the deer population. Only after implementation of our DDP approach did we see an appreciable drop in the CMA deer population. Combined, over nine years, our efforts removed nearly 750 deer from our core management area of <1,000 ha demonstrating the effort required to locally manage open deer populations. In some years, we lethally removed as many deer as we estimated existed in our core management area (Figure 8) highlighting the importance of deer dispersal and deer foraging. Populations quickly rebounded (our population estimation occurred before fawning season), although the long‐term trajectory is showing declines despite persistent immigration.

Since their establishment in the early 1900s, state wildlife agencies have been able protect and recover deer populations in North America to historically high levels. However, they are financially and philosophically poorly equipped to effectively address current conservation challenges associated with negative impacts of high deer populations (Jacobson, Organ, Decker, Batcheller, & Carpenter, 2010). Ecological or human health concerns have minimal impact on decisions about desirable deer population goals, in part, because management agencies do not implement routine assessments of ecological health indicators to guide deer management decisions, and thus such (unrecognized) impacts cannot inform public attitudes or management decisions (Riley et al., 2002). Further complicating the issue is that deer impacts are not necessarily a function of deer abundance or density, the metric often used to define landscape‐level population management goals (Putman, Watson, & Langbein, 2011). Despite repeated calls to adopt accountability and good governance principles in more holistic stewardship and wildlife management (Decker et al., 2016; Hare & Blossey, 2014; Leopold et al., 1947), agencies continue to focus largely on interests of stakeholders who buy hunting and fishing licenses. Our own experience and the overwhelming scientific evidence for the primary role of deer in the deterioration of ecological, economic, and health of our landscapes in the presence of recreational hunting (Côté et al., 2004; Kelly, 2019; Kilpatrick et al., 2014; Miller & McGill, 2019; Nuttle et al., 2011; Raizman et al., 2013) does not bode well for the future, unless major changes are implemented.

Restoring and maintaining diverse and healthy landscapes into the future will require, first and foremost, changes in deer management. We have no evidence that this can be accomplished using recreational hunting. In the past, strong winters caused major deer mortality in traditional winter yards, however, with climate change and milder winters with less snow cover, this deer mortality is no longer a major mortality factor. Use of regulated market hunting may be an important tool in the immediate future (Vercauteren et al., 2011). We further believe that healthy landscapes require top predators (Estes et al., 2011) and argue that species such as mountain lions and wolves should be afforded federal protection and be allowed to return and recolonize their traditional ranges across the continent. Through their consumptive effects and the creation of a landscape of fear, we anticipate cascading effects that will benefit not just primary producers but a beneficial restructuring of entire food webs (Clinchy, Sheriff, & Zanette, 2013; Manning, Gordon, & Ripple, 2009; Suraci, Clinchy, Dill, Roberts, & Zanette, 2016). We recognize that this is currently highly controversial in North America, but Europe is leading the way in trying to restore large terrestrial predator communities (Chapron et al., 2014). Regardless what options are implemented, the development of indicators or metrics to gauge deer impacts and to determine how changes in deer management affect the health of ecosystems and people is paramount. Society will need to decide how to fund regular assessments, and whether the responsibility for implementation of assessments will rest solely with wildlife management agencies. But managing wildlife as a public trust resource demands that all citizens will have the ability to obtain regularly updated information about the status of land health, and hold management agencies accountable if performance is lacking (Hare & Blossey, 2014).

Our oak sentinel approach showed great promise as an assessment tool. A large number of methods and metrics have been proposed to assess deer impacts, including plant community composition (Habeck & Schultz, 2015), woody browse indices (Morellet, Champely, Gaillard, Ballon, & Boscardin, 2001; Pierson & DeCalesta, 2015; Waller, Johnson, & Witt, 2017), and performance (height and flowering) of herbaceous species (Balgooyen & Waller, 1995; Fletcher, McShea, Shipley, & Shumway, 2001; Williams, Mosbacher, & Moriarity, 2000). Woody browse indices fail to measure impacts on herbaceous species, and other methods require presence of existing specimens. In areas with long‐existing large deer populations and depauperate landscapes, these species may no longer be present. By not relying on existing seedlings, saplings, or herbaceous plants that may differ in composition, age, or abundance among sites, we were able to standardize assessment protocols across sites and years. As such, our methodology is applicable at the local and regional scale and allows rapid assessment (within 100 days) of local deer browsing pressure helping managers rapidly evaluate outcomes following potential changes in deer management regulations or approaches. Under low deer browsing pressure, *Q. rubra* seedling mortality is low (20% over a 6‐year period in Wisconsin) and 3% per year in the southern Appalachian Mountains, although annual mortality for slow growing individuals may increase to 10%–15% (Kaelke et al., 2001; Wyckoff & Clark, 2002). Annual *Q. rubra* seedling browse rates exceeding 10%–15% are unlikely to enable regeneration in a species needing a decade or longer to grow sufficiently tall to place the top leader out of danger of being browsed by deer. However, we likely need to reduce acceptable rates of oak seedling browse even further if we want to protect more sensitive plant species. Herbaceous species, such as *Trillium grandiflorum* or *T. erectum*, continue to suffer browse rates that will lead to local extinction (Knight et al., 2009), even in areas where browse rates of oak seedlings fall below 15% (B. Blossey, unpublished data).

Due to its ease of implementation and the demonstrated sensitivity to changes in the size of the deer population, we believe oak sentinels are an important tool in assessing landscape health. We recognize that oak sentinels alone will not suffice and that additional more browse‐sensitive indicator species will need to be developed to allow assessments once deer populations have declined. Holistic management will also require that additional ecological, social, human health, and economic metrics will be required to create a portfolio of indicators that can guide decision making in holistic deer and landscape management. The future of our forests, the biodiversity contained in them, climate change mitigation, and human health are closely linked to our ability to embrace the required changes in deer management.

## CONFLICT OF INTEREST

No conflict of interest exists with the submission of this manuscript.

## AUTHOR CONTRIBUTIONS

BB developed the oak study; PC and JB developed the deer project; AD analyzed the data; and BB led the MS writing to which all authors contributed.

## Data Availability

Data associated with this work are available in the Dryad Digital Repository: http://doi.org/10.5061/dryad.6q573n5v5.
