# Supplementary material for: Red oak seedlings as indicators of deer browse pressure: Gauging the outcome of different white‐tailed deer management approaches
Source: Ecol Evol. 2019 Nov 8;9(23):13085–103. doi: 10.1002/ece3.5729 (PMC6912884; doi:10.1002/ece3.5729)
Supplement: Supplementary file 1 [file ECE3-9-13085-s001.docx]

**Blossey et al: Oak sentinels**

**Table S1.** Results for Mixed Effects Cox Regression models evaluating the effects of management regime (MR, no management, hunting or sterilization), fencing (F, fenced or open), initial oak height (IH) and mean vegetation height at time of planting (VH) on oaks planted at 15 sites in 2010 (A) and at 14 sites in 2011 (B). All models included site as random effect. Models are ordered according to lowest AIC value. dAIC is the difference between the AICc of a given model compared to the lowest AICc observed. The Akaike weight is calculated as the model likelihood normalized by the sum of all model likelihoods; values close to 1.0 indicate greater confidence in the selection of a model. Only models within 2 AICc are considered.

1. 2010

| **Factors** | **df** | **LogLik** | **AIC_c_** | **ΔAIC_c_** | **Weight** |
| --- | --- | --- | --- | --- | --- |
| MR * F + VH | 16 | -1650.37 | 3334.9 | 0 | 0.66 |
| MR * F + VH + IH | 17 | -1650.15 | 3336.5 | 1.63 | 0.29 |
| MR + F + VH | 14 | -1655.13 | 3340.1 | 5.26 | 0.05 |
| MR * F * IH + VH | 22 | -1648.84 | 3344.4 | 9.55 | 0.006 |
| MR * F * IH * VH | 24 | -1648.20 | 3347.4 | 12.58 | 0.001 |
| MR * F | 15 | -1661.51 | 3354.4 | 19.51 | 0 |
| MR * F + IH | 16 | -1661.1 | 3355.6 | 20.76 | 0 |

1. 2011

| **Factors** | **df** | **LogLik** | **AIC_c_** | **ΔAIC_c_** | **Weight** |
| --- | --- | --- | --- | --- | --- |
| MR + F + IH + 2^nd^ order interactions | 18 | -2037.0 | 4111.6 | 0 | 0.32 |
| MR * F + IH | 15 | -2040.1 | 4111.8 | 0.14 | 0.30 |
| MR * F | 14 | -2041.6 | 4112.7 | 1.12 | 0.18 |
| MR * F + IH | 13 | -2042.5 | 4114.3 | 2.66 | 0.09 |
| MR + F | 12 | -2044.8 | 4114.9 | 3.25 | 0.06 |
| MR * F * IH | 20 | -2036.8 | 4115.4 | 3.75 | 0.05 |

**Table S2.** Competing risk analysis for deer herbivory, rodent attack and unknown mortality occurring in presence of competing factors on oaks planted at 15 sites in 2010 (A) and at 14 sites in 2011 (B). Models are ordered according to lowest AIC value for 2010. dAIC is the difference between the AICc of a given model compared to the lowest AICc observed. The Akaike weight is calculated as the model likelihood normalized by the sum of all model likelihoods; values close to 1.0 indicate greater confidence in the selection of a model. Only models within 2 AICc are considered. Vegetation height was not evaluated in 2011.

|  | 1. **2010** | | | |  | 1. **2011** | | | |
| --- | --- | --- | --- | --- | --- | --- | --- | --- | --- |
| 1. **Deer herbivory** |  |  |  |  |  |  |  |  |  |
| Factors | df | LogLik | AIC_c_ | ΔAIC_c_ |  | df | LogLik | AIC_c_ | ΔAIC_c_ |
| Management regime | 2 | -1024.4 | 2052.8 | 0 |  | 2 | -1015.5 | 2034.9 | 0 |
| Management regime + vegetation height | 3 | -1023.9 | 2053.7 | 0.93 |  |  |  |  |  |
| Null | 0 | -1030.9 | 2061.8 | 9.0 |  | 0 | -1021.9 | 2043.9 | 8.97 |
|  |  |  |  |  |  |  |  |  |  |
| 1. **Rodent attack** |  |  |  |  |  |  |  |  |  |
| Factors | df | LogLik | AIC_c_ | ΔAIC_c_ |  | df | LogLik | AIC_c_ | ΔAIC_c_ |
| Management regime | 2 | -265.68 | 535.36 | 0 |  | 2 | -71.34 | 146.68 | 3.30 |
| Management regime + vegetation height | 3 | -264.77 | 535.54 | 0.18 |  |  |  |  |  |
| Null | 0 | -276.20 | 552.39 | 17.02 |  | 0 | -71.70 | 143.38 | 0 |
|  |  |  |  |  |  |  |  |  |  |
| 1. **Unknown mortality** |  |  |  |  |  |  |  |  |  |
| Factors | df | LogLik | AIC_c_ | ΔAIC_c_ |  | df | LogLik | AIC_c_ | ΔAIC_c_ |
| Null | 0 | -42.87 | 85.74 | 0 |  | 0 | -243.94 | 487.89 | 0 |
| Management regime | 2 | -43.42 | 88.83 | 3.10 |  | 2 | -241.95 | 487.91 | 0.02 |
| Management regime + vegetation height | 3 | -42.10 | 90.20 | 4.44 |  |  |  |  |  |

**Table S3.** Annual proportion of unprotected oaks browsed by deer during the growing season (June-October) at different sampling locations in different years and the estimated deer abundance in the core management area (N=20 oaks per location).

| **Site** | **Sampling year** | | | |
| --- | --- | --- | --- | --- |
|  | **2010** | **2011** | **2014** | **2015** |
| Dyce Lab | 0.65 | 0.50 | 0.10 | 0.20 |
| Golf Course | 0.70 | 0.70 | 0.15 | 0.20 |
| Hungerford Hill |  |  | 0.20 | 0.30 |
| McGowan Woods | 0.50 | 0.35 | 0.05 | 0.05 |
| Palmer Woods |  | 0.45 | 0.25 | 0.20 |
| Slim Jim Woods | 0.50 | 0.65 | 1.00 | 0.80 |
| Warren Woods | 0.50 | 0.25 |  | 0.20 |
| Mean browse rate (±SEM) | 0.57(0.04) | 0.48(0.07) | 0.29(0.15) | 0.28(0.09) |
| Mean deer abundance (95%CI) | 99 (89-110) | 89 (78-101) | 58 (46-73) | 47 (36-61) |
